# Understanding pharmacists' engagement in sport and exercise medicine, including pharmacist-physiotherapist collaboration: A qualitative study and COM-B analysis.

**DOI:** 10.1016/j.rcsop.2025.100593

**Published:** 2025-03-20

**Authors:** Alison D. Hooper, Jodie Marquez, Beata Bajorek, Joyce Cooper, David Newby

**Affiliations:** a School of Biomedical Sciences & Pharmacy, College of Health, Medicine & Wellbeing, University of Newcastle, Callaghan, NSW, Australia; b School of Health Sciences, College of Health, Medicine and Wellbeing, University of Newcastle, Callaghan, NSW, Australia; c College of Medicine & Dentistry, James Cook University, Cairns, QLD, Australia

**Keywords:** Sports pharmacy, Sport and exercise medicine, Pharmacists, Interprofessional collaboration, Physiotherapy, COM-B model, Behaviour change

## Abstract

**Background:**

Sport and exercise medicine (SEM) is a multidisciplinary field that integrates expertise from various healthcare professionals to optimise athletic performance and promote physical activity for chronic disease prevention and management. Australian pharmacists are well-positioned to contribute to SEM, yet their roles remain undefined beyond niche areas like anti-doping. Interdisciplinary collaboration, particularly with physiotherapists, is also underexplored. This study investigates pharmacists' engagement in SEM and pharmacist-physiotherapist collaboration, using the Capability, Opportunity, Motivation–Behaviour (COM—B) model to explore behavioural components.

**Methods:**

A qualitative study was conducted using semi-structured interviews with 14 Australian pharmacists practicing across diverse settings. Data were thematically analysed and mapped to the COM-B framework.

**Results:**

Five key themes emerged: (1) Broad scope of pharmacy practice in SEM incorporating both pharmacological and non-pharmacological advice; (2) Opportunities and challenges in inter-professional collaboration, constrained by informal referral pathways and limited interdisciplinary communication (3) Gaps in SEM-related training and education, with pharmacists expressing interest in targeted professional development; (4) Perceived barriers to engagement, including time constraints, remuneration issues and lack of professional recognition; and (5) Future opportunities for pharmacists in SEM, such as integration into multidisciplinary SEM teams and supporting physiotherapist prescribing.

**Conclusions:**

Pharmacists are well-placed to play a broader role in SEM but face systemic and educational barriers. Enhancing training, establishing formal referral and interdisciplinary communication pathways and addressing structural challenges could improve engagement. This study lays the groundwork for future interventions to enhance pharmacists' contributions to SEM and strengthen pharmacist-physiotherapist collaboration, ultimately improving consumer care and health outcomes.

## 1 Background

Sporting culture is often considered quintessential of the Australian way of life. Sport and exercise medicine (SEM) is a discipline that has evolved to encompass a multidisciplinary approach that encompasses both optimisation of sporting performance at all levels, as well as the promotion of physical activity in the prevention and management of chronic diseases such as arthritis, diabetes and cardiovascular disease.^1^ Thus, consumers of SEM are not only competitive athletes, but also recreationally active individuals as well as those who stand to benefit from increased physical activity in managing or preventing chronic conditions.1., 2., 3., 4., 5.

Contemporary SEM is inherently multidisciplinary, integrating expertise from various health disciplines.^6^^,^^7^ As trusted and accessible healthcare providers, Australian pharmacists are well-positioned to contribute to this broader SEM framework.^8^ The National Competency Standards Framework for Pharmacists (2016) identifies sport- and exercise-related healthcare and advice as within their professional scope.^9^ Yet, roles for pharmacists in SEM have not been comprehensively described. Existing research and limited training opportunities in ‘sports pharmacy’ primarily focus on niche areas, such as advising on doping prevention and control in elite or competitive sports.9., 10, 11, 12, 13 However, contemporary SEM takes a far broader view.^1^^,^^4^^,^^7^^,^14., 15., 16, 17., 18. This gap leaves significant potential for pharmacists to engage in SEM more comprehensively. Notably, no Australian studies have explored pharmacists' perceptions of their roles within the multidisciplinary SEM context.

SEM has the potential to be practiced across various healthcare settings, with pharmacists engaging in different capacities depending on their workplace environment. While no research has comprehensively explored pharmacists' roles in SEM across settings, hospital and elite sports environments may provide greater access to multidisciplinary teams. In contrast, community pharmacists frequently serve as the first point of contact for SEM-related queries from the general public, given their accessibility and widespread presence. Despite this, pharmacists' roles in SEM remain largely undefined across all settings.

In contrast, physiotherapists have well-established and recognised roles in SEM and are frequently the first point of contact for consumers seeking care.^7^^,^^19^ Beyond managing sports-related injuries, physiotherapists may also be called upon to provide advice to consumers about the use of medicines and supplements.^20^, ^21^, ^22^ However, professional challenges have been identified, particularly for those practicing in areas without adequate medical or pharmacist support.^22^^,^^23^ The Australian Physiotherapy Association has advocated for autonomous prescribing for registered physiotherapists while emphasising the importance of collaboration with pharmacists to ensure safe and effective use of medicines.^24^ Despite these developments, research has yet to describe pharmacist-physiotherapist inter-disciplinary collaboration in delivering holistic SEM care in Australia underscoring the need to better define and integrate these roles within the SEM landscape.

Understanding what influences pharmacists' engagement within the multidisciplinary SEM landscape is essential to optimising SEM healthcare delivery. Conceptualising pharmacists' practice in SEM as a behaviour may aid in identifying enablers and barriers to their engagement. Behaviour change interventions often fail because of incorrect assumptions about what needs to be addressed.^25^^,^^26^ Improving or changing healthcare practices and behaviours requires a comprehensive understanding of their underlying dynamics, as these behaviours do not occur in isolation; they exist within a broader system, interacting with intra- and inter-individual factors.^26^

The COM-B (Capability, Opportunity, Motivation, and Behaviour) model provides a framework to explore behaviour and its components within a specific context, functioning as an interconnected system. Researchers and practitioners have extensively used the COM-B model to understand the behaviour of pharmacists and other health professionals.^27^, ^28^, ^29^, ^30^ This information can be used to inform the development of behaviour change interventions using the Behaviour Change Wheel (BCW).^26^

To date, the engagement of Australian pharmacists in SEM, including their collaboration with physiotherapists, has not been conceptualised, nor have the factors influencing their practice within SEM been identified. This study aimed to explore Australian pharmacists' perceptions and experiences about sports pharmacy, using the COM-B model as an analytical framework for understanding the enablers and barriers to their engagement in SEM. The specific objectives were to explore the scope of pharmacists' practice in relation to SEM, behavioural components influencing pharmacists' engagement in SEM and to describe the degree and nature of pharmacist-physiotherapist inter-disciplinary collaboration in SEM. A better understanding of these aspects of practice and behaviour will help inform targeted interventions to enhance pharmacists' contributions to SEM in the future.

## 2 Method

### 2.1 Study design

Given the limited exploration of pharmacists' roles within the broader context of contemporary SEM in the published literature, and the exploratory nature of the study, a qualitative study design was chosen to gain a deeper understanding of pharmacists' practices in SEM from the perspective of respondents.^31^ The study, grounded in behavioural theory, employed semi-structured interviews with Australian pharmacists to explore their engagement in SEM, including collaboration with physiotherapists. The consolidated criteria for reporting qualitative studies (COREQ) guided the reporting of this study.^32^

### 2.2 Ethics approval

Ethics approval for the study was granted by the institutional Human Research Ethics Committee (Protocol number: H-2020–0075).

### 2.3 Theoretical framework

**Fig. 1:**
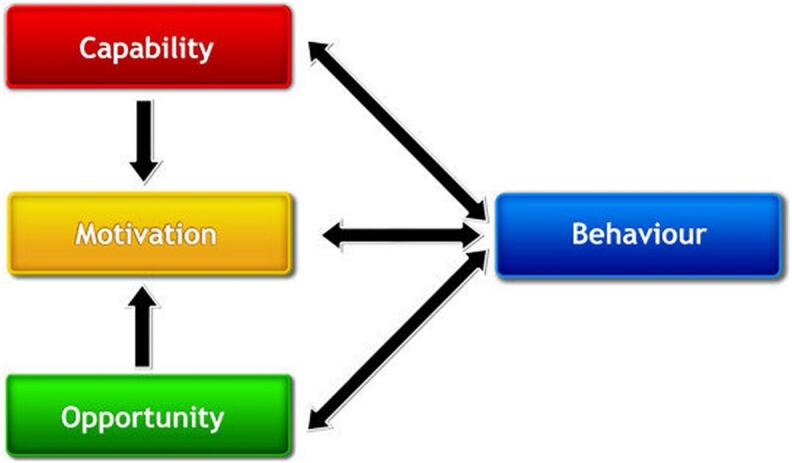
The COM-B model of behaviour. Michie S, Atkins L, West R. (2014) The Behaviour Change Wheel: A Guide to Designing Interventions. London: Silverback Publishing. www.behaviourchangewheel.com.

**Fig. 2:**
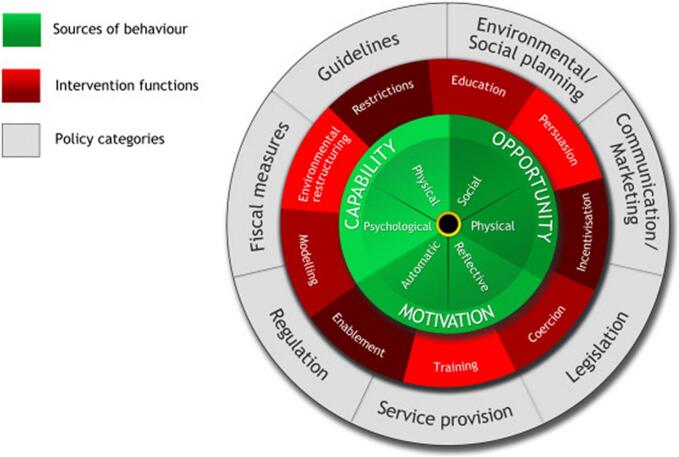
The Behaviour Change Wheel. Michie S, Atkins L, West R. (2014) The Behaviour Change Wheel: A Guide to Designing Interventions. London: Silverback Publishing. www.behaviourchangewheel.com.

This qualitative study is grounded in the Behaviour Change Wheel (BCW) framework, with the COM-B model of behaviour at its core, serving as the primary theoretical underpinning (Fig. 1, Fig. 2 respectively). The COM-B model was utilised in this study for two key purposes: to inform the development of the interview guide and to analyse and contextualise the data.^25^^,^^26^^,^^33^ The choice of the COM-B model was driven by its comprehensiveness and flexibility for analysing behaviours across various contexts.^25^^,^^26^^,^^33^ By incorporating the COM-B model during both the instrument design and data analysis phases, the study ensured alignment with a robust theoretical foundation that systematically addresses behaviour through the lens of capability (physical and psychological), opportunity (social and physical) and motivation (reflective and automatic). The explicit integration of the theoretical framework into both the method and analysis enhances the study's rigour and ensures the findings are firmly rooted in an evidence-based approach to understanding behaviour.

The choice of the COM-B model was motivated by its comprehensiveness and adaptability for examining behaviours across diverse contexts. It has been widely applied to explore barriers and facilitators in healthcare delivery. Examples include studying hand hygiene practices among healthcare professionals,^34^ investigating barriers and facilitators in providing diet and physical activity advice to young mothers,^35^ and examining healthcare professionals' barriers and facilitators to delivering opportunistic behaviour change interventions during routine medical consultations.^36^

### 2.4 Setting and participants

The study was conducted in Australia which has a multi-cultural, dual-sector healthcare system whereby healthcare is either government-subsidised or privately funded. The target participants were Ahpra-registered (Australian Health Practitioner Regulation Agency^37^) pharmacists practicing in an Australian healthcare setting, meeting the following inclusion criteria: having access to a reliable internet connection for participating in an online interview via a videoconferencing platform. Purposive and convenience sampling, with a subsequent snowballing approach, were used to achieve maximal variation in recruiting participants, including pharmacists from both rural and remote, and urban areas, to capture varying experiences in SEM.

An invitation to participate in the research was sent via email; contact details of potential participants were accessed from publicly available websites (pharmacy business web pages) identified via Google searches for ‘pharmacy’. Individuals who wished to participate could indicate their intention to do so via the email address provided on the study information. Individuals who expressed interest in participating in the study were invited to provide an email address to receive an electronic copy of the Participant Information Statement, which provided details of the research study, researchers' background and reasons for undertaking the research, and Consent Form. Snowballing was encouraged, whereby initial ‘contacts’/potential participants were invited to pass the Study Information on to business or personal contacts. Written and recorded consent was obtained from participants prior to the interviews.

### 2.5 Data collection

A mutually convenient date/time for each interview was arranged via email. All interviews were conducted online and audio recorded via Zoom; this was initially due to social restrictions brought about by the COVID-19 pandemic, and later for consistency. All interviews were conducted by AH with only AH and the participant present for each interview to encourage open discussion. Interviews were recorded and interview data was supplemented by note-taking during and immediately following the interviews.

#### 2.5.1 Interview guide

An interview guide aligned to the stated research objectives was constructed with input from members of the research team, drawing on the theoretical framework, literature review^10^ and expert opinion (Table 1, Appendix A). Demographic questions were incorporated into the interview guide to collect baseline data on practice location, years registered and post-graduate qualifications.

Interview questions were informed by a literature review on pharmacists' roles in SEM, including the results of a systematic review exploring current and potential roles in sports pharmacy, and prompts were developed based on the elements of the COM-B model. Interview questions were aligned to the stated research objectives.

### 2.6 Researcher characteristics and training

AH (female) is a PhD candidate and holds dual qualifications as a pharmacist and physiotherapist, holding a Master of Pharmacy and a Bachelor of Physiotherapy degree. AH has 15 years' experience as a pharmacist and academic in Australia, 2 years' experience as a registered physiotherapist and has an academic interest in sport and exercise medicine, fueled by her dual professional background. DN, TK, JC and BB are registered pharmacists, academics and experienced pharmacy practice researchers. JM is a registered physiotherapist, academic and experienced researcher.

BB has extensive experience in applying behaviour change theories, and DN, TK, JC, BB and JM all have considerable experience in qualitative methodology.

TK provided training to AH in conducting interviews. The training involved in-depth discussions about research topics and objectives, guidance on facilitating semi-structured interviews and debriefing sessions following pilot interviews. Additionally, TK offered training on coding and thematic analysis, covering various coding techniques, both inductive and deductive, and providing feedback on the coding of pilot transcripts. AH also undertook training in qualitative coding and thematic analysis using NVivo software.

### 2.7 Piloting

AH conducted two pilot interviews, testing videoconferencing and transcription technology. Minor adjustments were subsequently made to the interview questions for clarity and additional prompts.

### 2.8 Data analysis and presentation

*Qualitative coding and thematic analysis:* Interviews were transcribed *verbatim* for data analysis; grammatical flaws were retained to reflect context. Transcripts were not returned to participants for review or comment. Transcripts were qualitatively coded with the aid of NVivo software (version 12), then thematically analysed using an inductive approach. To establish an initial list of codes based on emergent concepts, the first two interviews were systematically coded line-by-line. This provided a coding list for each consecutive transcript, with any new emergent codes added as required . Already coded transcripts were reviewed for retrospective fit of new codes. These codes were grouped into sub-themes and themes.

*COM-B model mapping:* Following the thematic analysis, the emergent themes were then mapped to the COM-B model to identify the components of behaviour influencing pharmacists' engagement in SEM. This two-step analysis involved pulling out data that specifically addressed elements of Capability, Opportunity and Motivation.

Secondary analysis was performed by an experienced researcher (independent of the research team) on all transcripts. The primary and secondary analyser convened to achieve coding congruency, resolving any discrepancies, and then discussed themes and COM-B categorisation until agreement was achieved.

The data analysis process maintained methodical transparency, allowing an audit trail from the final interpretation of themes back to the original raw data transcripts, and the coding process was discussed by the research team throughout data analysis. Data analysis was conducted while data collection was still underway to allow for identification of data saturation. The point of data saturation was determined retrospectively as the point at which no new themes emerged from the data from at least two consecutive interviews. Once data analysis revealed thematic saturation, no further interviews were scheduled.

Illustrative quotes are provided verbatim for authenticity. Quotes are labeled according to participant number (Pharm 1–14), followed by practice setting and practice location. Practice setting: CP = community pharmacy, CH = both community and hospital, CM = both community and home medicines review, PS = combination of professional sport, community and hospital. Practice location: MO = metropolitan only, RR = rural/remote, MR = both metropolitan and rural/remote.

### 2.9 Rigour and trustworthiness

A semi-structured interview guide allowed for standardisation of core interview questions whilst allowing conversational flexibility for deeper exploration of emergent concepts. Transcripts were independently analysed by two researchers, with interpretations of the data reviewed collaboratively by members of the research team. This process, involving theme validation through peer review and researcher triangulation, ensured consistency and agreement on the identified themes, reduced individual bias, and strengthened the rigour of the analysis.^38^, ^39^, ^40^While respondent validation can further support data credibility, they were not undertaken in this study to maintain participant confidentiality, as the study protocol restricted further contact with participants post-interview.

## 3 Results

Fourteen interviews were conducted between March 2020 and July 2024. Interviews lasted between 20 and 35 min. Upon retrospective analysis, data saturation was reached by the 8th interview. Scheduled interviews were completed, but no further appointments were arranged.

### 3.1 Participant characteristics

Participants had diverse professional backgrounds, practice settings and levels of experience. Most worked in community pharmacy, with others in mixed settings or professional sports. Practice locations varied between urban, rural and mixed areas. Participant demographic data is summarised in Table 2.

**Table 2:** Participant demographic data.

Category	Sub-category	Value
Total number of participants	–	14
Years registered: mean (range)	–	16.5 (1.5–32)
Gender	Male	2
	Female	12
Practice setting	Community	9
	Both community and hospital	2
	Both community and home medicines review	2
	Combination of professional sport, community and hospital	1
Practice location (self-identified)	Metropolitan only	10
	Rural/remote	3
	Both metropolitan and rural/remote	1
State of practice	New South Wales	11
	Queensland	2
	Victoria	1

### 3.2 Thematic analysis

Five key themes emerged from the analysis, reflecting experiences and perceptions related to engagement in SEM and collaboration with physiotherapists:

1. Broad scope of pharmacy practice in SEM incorporating both pharmacological and non-pharmacological advice;
2. Opportunities and challenges in interprofessional collaboration in SEM;
3. Training and education;
4. Perceived barriers to engagement in SEM;
5. Future opportunities for pharmacists in SEM.

Themes and sub-themes are presented in Table 3 (Appendix B), mapped to components of the COM-B model and categorised as barriers or enablers.

### 3.3 COM-B model mapping

#### 3.3.1 Theme

**1: Broad scope of pharmacy practice in SEM incorporating both pharmacological and non-pharmacological advice.**

**Capability (Physical, Psychological)**

- **Pharmacists as medicines experts**

All pharmacists expressed confidence in their expertise as medicines specialists, identifying this as their primary role in SEM. They particularly highlighted their proficiency in applying their understanding of pharmacological interventions to the SEM context, such as the appropriate use of anti-inflammatory and analgesic medications. *– Capability (Psych).*

*“I find I find that I'm most confident giving advice in and around medicines that I'm more familiar with for other purposes. So if someone has, you know, a mild injury requiring pain relief for example or first aid around, you know, rest and elevation and all that kind of thing, I find that I'm more confident, you know, recommending something like ibuprofen, because I'm familiar with the use of that drug, you know, in other therapy contexts as well.” (Pharm 2, CM, MO).*

When responding to queries about drugs in sports, some participants reported feeling constrained by time and uncertainty about where to find information, although they had some familiarity with accessing resources on anti-doping. They expressed a desire for more training opportunities to enhance their ability to make informed recommendations about drug use in sports. *– Capability (Psych).*

*“I know that some of our drug resources like MIMS have some limited information and lists that we can access but beyond that I was sort of more just going off my own bat in terms of where I might start to look.” (Pharm 2, CM, MO).*

Some respondents also viewed their role in SEM as incorporating advice around the effects of polypharmacy on physical capacity. They suggested that this aspect of their role could be better recognised within the multidisciplinary SEM team in the future. *– Capability (Psych).*

*“When we look at the more elderly patients, looking at their medications before they get to the exercise physiologist the first time, because obviously a lot of their medications can affect how the body's going to respond to, for example, blood pressure monitors and pulse rate, you know, if someone's going, ‘you have to push harder, push harder’ your pulse is not getting high enough, your heart rates, not getting high enough, and they're on a beta blocker (…)it's not going to get high.”*

*(Pharm 5, CP, MO)*

- ***Broad scope of queries in SEM***

All pharmacists viewed their role in SEM as encompassing a wide range of responsibilities and expertise. Perceived scope ranged from medicines advice and injury prevention and management to chronic disease management and prevention through physical activity. A list of the services and products participants perceived as being within the scope of a pharmacist's role in SEM is provided in Table 4. *– Capability (Phys, Psych)*

**Table 4:** Services and products perceived to be within the scope of the pharmacists' role in SEM.

**Services**	**Products**
Recommendations and advice to consumers regarding medicines Advice about medicines safety (e.g. drug-drug interactions, drug-supplement interactions, adverse effects) Advice about doping prevention and control Provision of non-pharmacological advice about minor injuries (e.g. strains, sprains) Provision of advice about the prevention and management of minor ailments related to participation in sport and physical activity (e.g. swimmer's ear, cramps, dehydration, aches and pains) Roles in sporting contexts ranging from local club sports to international competitions such as the Olympic and Paralympic games Provision of advice about stocked products Provision of advice about nutrition First aid Wound care Triage and referral Travel medicine (e.g. vaccinations) Education of SEM healthcare providers, consumers and other key stakeholders Health promotion across the lifespan Support for prescribers on drug interactions, medicines safety, dosing, legislation and prescription requirements Comprehensive medication management review Provision of pharmacotherapeutic advice to support off-label prescribing in professional sports Chronic disease prevention and management Specialised compounding services Promotion of sport and physical activity in the community (e.g. partnerships with local sporting clubs and walking groups) Provision of education and advice to players, coaches and parents of local sporting clubs	Over-the-counter medicines Supplements Prescription medicines Strapping tape Braces Mobility aids (e.g. crutches, walkers) Ice and heat packs Mouth guards First aid kits and products Electrophysical devices (e.g. TENS machines) Foot care (e.g. shoe inserts) Oral rehydration

- **Acting as a triage point**

While triage this was identified as a key role for pharmacists, limited knowledge of referral pathways was seen as a barrier. *– Capability (Psych).*

*“I think that that is a very, very important aspect of pharmacy in terms of the triaging roll and the capability to appropriately refer. I don't necessarily think that pharmacists are trained on how to refer and who to refer to, so we're kind of in the dark, I guess, in a sense to say ‘oh, hey, I think you need to see a physiotherapist, but I'm not completely sure, or within the time frame that's appropriate,’ for example. You know, do they need to go and see someone that day or is it something that they maybe can see in a couple of days' time.” (Pharm 1, CP, MO).*

### 3.4 Opportunity (Physical, Social)

- **Frequent SEM-related queries**

Pharmacists described frequent interactions with consumers seeking SEM services. *- Opportunity (Phys, Soc).*

*“We get a lot of queries over the counter of it with respect to sports medicine... A lot of musculoskeletal injuries. So related to ankles, knees. Heat packs. That's a big one as well. A lot of questions around that... I also get a lot of sports nutrition questions so related to vitamin supplements perhaps kind of various proteins that are available. A lot of, I guess, I do often get children as well.” (Pharm 1, CP, MO).*

Some participants reported more frequent presentations in community pharmacies on weekends when other healthcare services may be closed, citing challenges associated with limited resources supporting pharmacists in provision of primary care SEM services. *Opportunity (Phys).*

*“Where's that resource for me on a Saturday morning or a Sunday morning to go: ‘I can help you’ when nothing else is open.”*

*(Pharm 11, CP, MO)*

- **Broad scope of queries in SEM**

All pharmacists perceived the scope of pharmacists' roles in SEM as broad, encompassing both pharmacological and non-pharmacological advice and products. Queries about medicines use were seen to be most frequent, which not only included the effective and safe use of medicines, but also advice about prohibited substances in sport. Medicines-related queries included advice about pain relief following minor sporting injuries, medicines for minor sporting-related ailments, facilitation of injury recovery and return-to-sport, specialised compounding services and advice about whether a drug is prohibited in a particular sport. *– Opportunity (Phys; Soc).*

*“I'm confident with, sort of, the medicines in terms of like if it was pain relief, you know, if it was something like swimmers ear, because those are common things.” (Pharm 2, CM, MO).*

*“We do have some more serious competitors, including the Paralympic competitors, that come in wanting to, getting prescriptions and wanting to make sure that it's actually okay to use those medications in their sport when they are potentially going to be tested.” (Pharm 5, CP, MO).*

*“A couple of examples would be pain relief following what is mainly minor sporting injuries. And also, oh yes, prohibited substances. So elite athletes, yeah, prior to competition, they might inquire about whether the substance that they're getting on prescription - so it might be a drug that they're not using in the line of their the sport or anything to do with their sport, but whether that drug - would have implications on their sport. So I guess one of the most common ones I've had is in and around the use of inhalers (…) I had a recent customer who I did a home medication review for, and he was in the (…) bodybuilding or weightlifting type area, and he suffered a myocardial infarction (…) he'd previously not been on any medicines and then he subsequently ended up on a range of new medicines. He was particularly concerned about some of the beta blockers and their existence on the anti-doping lists etc., because that would preclude him from doing his sport competitively.” (Pharm 2, CM, MO).*

*“We often do a compounding product that will actually, you know, get through the dermal layers, it will actually provide proper transdermal delivery.” (Pharm 9, PS, MO).*

A broad range of queries from elite sporting contexts to recreational physical activity as well as physical activity for chronic disease management and prevention was highlighted. *– Opportunity (Phys, Soc).*

*“There's a lot of older people that come in. Maybe the gardening or walking or swimming even, you know, doing some exercise for their osteoarthritis pain and then they've maybe exacerbated that somehow. That's pretty common.” (Pharm 3, CP, MO).*

*“Highlighting all the areas of need for athletes, whether that be in a social context or in a more competitive context, and then tailoring services around those needs and ensuring that pharmacists are upskilled to be able to provide those services. I personally put my name down when the Commonwealth Games were on the Gold Coast to see if I could go up and, yeah, be in the pharmacy up there for a portion of that.” (Pharm 2, CM, MO).*

Provision of advice concerning nutrition and supplements was also a common theme across the majority of interviews. *– Opportunity (Phys, Soc).*

*“I also get a lot of sports nutrition questions, so related to vitamin supplements, perhaps kind of various proteins that are available.” (Pharm 1, CP, MO).*

Pharmacists' scope in SEM was described by all respondents as incorporating non-pharmacological advice and provision of products and devices. *– Opportunity (Phys, Soc).*

*“A recent query involves someone who wanted to use some strapping tape, but they wanted a product to put underneath that strapping tape to avoid the adhesive, yeah, being problematic. I've also received queries around things like the provision of mouth guards. I find that it's really demographic specific… More recently I've worked in a demographic where there's a lot more of a coastal lifestyle. I've had queries along the lines of the right type of earplugs to use for surfing, for example. I've also had queries around first aid and I've had queries around preventative measures. So using things like knee supports prior to football matches.” (Pharm 2, CM, MO).*

*“A lot of people don't actually know how to fit crutches correctly and just have one, you know, size, apparently that fits all. It may not obviously always be the case. Even just the walkers and things like that. That's another thing where we don't have a lot of training at university. And that could be another area where we can receive information about that that could really help everyone a lot.”*

*(Pharm 3, CP, MO)*

- **Partnerships with local sporting clubs**

Some pharmacists, particularly those working in rural areas, emphasised the potential for partnerships with local sporting clubs. Provision of education and advice to players, coaches and parents, as well as supply and advice around products, particularly relating to stocking first aid kits. Financial support for grass-roots sport was mentioned by several participants. Some participants reported that pharmacists should seek additional education in SEM to enhance credibility and support for local sporting clubs with which they have formed a partnership. *- Opportunity (Soc).*

*“We do quite a bit of first aid supplied to our local clubs and areas. So they come to us and say ‘what do we need,’ you know, or ‘we need this’ or ‘how do we do that’.”*

*(Pharm 8, CP, RR)*

- **Diverse demographic of consumers across the lifespan**

Pharmacists described delivering SEM services to consumers across various age groups, reflecting diverse health needs. – *Opportunity (Phys).*

*“We do have the older the older patients that are coming in or getting, like I said, physically active for the first time. And also the younger ones, like gym addicts. So there is, there is really quite, quite a lot of people different groups that come in.”*

*(Pharm 5, CP, MO)*

- **Pharmacists' accessibility supports their role in SEM**

The accessibility of community pharmacists significantly contributed to the frequency of consultations, as consumers frequently approached them as their initial point of contact for SEM-related issues. The convenience of obtaining healthcare and advice from a professional without needing an appointment or incurring a fee-for-service was seen as a key enabler and a common motivation for consumers seeking pharmacists' care in SEM. A wide range of stocked products was also viewed as a contributor. *- Opportunity (Phys).*

*“It's being so accessible, too. You know, we're it. You know. Whatever your problem, they, they'll come to us first.” (Pharm 11, CP, MO).*

Accessibility was occasionally seen as a concern, with some pharmacists feeling obligated to offer advice that might extend beyond their personal scope of practice or professional boundaries. *– Motivation (Refl).*

*“We're getting a lot of queries that are actually for other healthcare professionals, but due to us, being the most accessible healthcare professional. We sort of field them.”*

*(Pharm 14, CP, MO)*

- **Consumer expectations**

A frequent theme was that consumers expect pharmacists to provide evidence-based advice on SEM-related products and services, particularly concerning products stocked in a community pharmacy. However, pharmacists acknowledged gaps in their knowledge and ability to meet these expectations, despite acknowledging that consumers should be able to expect evidence-based advice on stocked products. *– Opportunity (Soc).*

*“I just don't think pharmacists should have things in their pharmacy and be selling those things without having the ability to understand when they do and don't need to be used and then how to use them correctly. So I just feel that there's probably a big limitation in terms of pharmacists' knowledge. As a pharmacist, it has always bothered me because I think, you know, when someone comes in and we've got that product on the shelf, there's an element of guilt that we feel if we can't fully provide the service.” (Pharm 2, CM, MO).*

Pharmacists emphasised their commitment to providing evidence-based advice, despite the limited resources available for pharmacists in SEM, especially regarding non-pharmacological interventions. This scarcity sometimes led them to seek information from less reliable sources, especially those practicing in rural and remote locations. However, they highlighted interprofessional learning as a potential solution to address this challenge. *– Motivation (Refl).*

*“I reckon they'd be really helpful in helping us understand rehabilitation better, and, like, what the new evidence would suggest in certain injuries. Even how to strap would be something that they could probably contribute as well. You know, I'll usually look up YouTube videos on how to strap someone's injury if I'm not personally aware of it.”*

*(Pharm 10, CP, MR)*

- **Acting as a triage point**

Most respondents viewed pharmacists' role in SEM as acting as a point of triage for SEM-related concerns, particularly in community settings. *- Opportunity (Phys).*

*“So it's a matter of triage and referring, or not.” (Pharm 8, CP, RR)*

### 3.5 Motivation (Automatic, Reflective)

- **Pharmacists more likely to engage in SEM if they have a personal interest in sports**

Pharmacists with personal experience or interest in sports described greater confidence and engagement in SEM. *– Motivation (Auto).*

*“My family's interest in gym, body building, etc. Then it's just knowledge that's come upon us because we're interested in that area, rather than anything that's been offered to us. Yeah, so it'd be more likely I'd walk over to somebody in that sports supplementation area or with an injury, and you know, take an interest, definitely.” (Pharm 11, CP, MO).*

### 3.6 Theme.

**2: Opportunities and challenges in interprofessional Collaboration in SEM.**

**Capability (Psychological)**

- **Medicine requests post-physiotherapist consultation**

A few pharmacists expressed concerns about the appropriateness of medication requests from consumers following physiotherapy consultations, highlighting the need for greater pharmacist input. *– Capability (Psych).*

*“I often get people that have come from the physio that have been taking Nurofen, or have been suggested to take Nurofen for their injury and, you know, you find out there on a whole range of cardiovascular medications, for example, which is definitely a role that pharmacists can play in this in this space.” (Pharm 1, CP, MO).*

### 3.7 Opportunity (Physical, Social)

- **Pharmacists often suggest consumers see a physiotherapist**

Some pharmacists reported regularly recommending patients see a physiotherapist for SEM-related injuries. *– Opportunity (Soc).*

*“I refer patients to physio on a daily basis in my practice.”*

*(Pharm 4, CM, MO)*

- **Informal referral pathways**

While some pharmacists, particularly those practicing in community settings, reported frequently referring patients to physiotherapists and other health professional such as podiatrists, these referrals are typically informal. Participants noted the lack of structured referral processes as a barrier to effective interprofessional collaboration. *– Opportunity (Phys, Soc).*

*“It could just (…) better referral pathways between physios and pharmacists, whereby if someone comes into the pharmacy with a complaint, then we have a good referral pathway to a physiotherapist and then vice versa, if someone presents to the physio but they need advice in and around analgesic use, or maybe medicines used within the context of, you know, doping problems, and they could refer to the pharmacist. I'm not aware of any good systems like that in place. If I had to refer someone to a physio I would just say ‘you need to see a physio.’ I wouldn't write a note and I wouldn't, kind of, yeah, like send that off.” (Pharm 2, CM, MO).*

*“Referral pathways are huge in this area - who to refer to appropriately, which, again, I'm not sure we've had all that many opportunities to learn about that gap... So I think that's definitely a bit of a grey area that we could certainly look to improve upon the certain referral criteria, who to refer to, and when.”*

*(Pharm 1, CP, MO)*

- **Enthusiasm for interdisciplinary collaboration**

Pharmacists with experience in hospital environments viewed this practice setting as more conductive to inter-professional collaboration in SEM. *– Opportunity (Phys).*

*“Quite often you'll have the dietician in the room, the physio, as well as whatever specialty doctors. So I think in that regard a hospital pharmacist is better prepared because they already know how to liaise with those colleagues.” (Pharm 9, PS, MO).*

Community settings where multiple practitioners were co-located lead to mixed perceptions about whether this co-location facilitated improved opportunities for collaboration. *– Opportunity (Phys).*

*“A lot of pharmacies are situated in practices where they're next to a doctor, next to a physio, and having that multi-disciplinary kind of area being physically located. So where I work, we're right next to the doctors, and then we've got a physio, and then some of the specialists as well, all in one kind of setting. So it's very easy to say ‘why don't you go and make an appointment’.” (Pharm 8, CP, MO).*

*“If you look at co-location sites like a pharmacy next to a GP practice that might have a physio, like, even they don't talk properly. Like, you know, it doesn't, yeah, for whatever reason, the communication doesn't happen.”*

*(Pharm 9, PS, MO)*

- **Medicine requests post-physiotherapist consultation**

A few pharmacists reported receiving medication requests following physiotherapy consultations. While collaboration was encouraged, there was perceived room for improvement in these requests and in enhancing collaboration with pharmacists. *– Opportunity (Soc).*

*“we do get a quite a few questions about, I guess, the physio relays information on to the patient and then on to us, and it may not be the most appropriate course of action because they don't always go into, you know, other medication, and they're taking things like that. So yeah, pretty common.” (Pharm 3, CP, MO).*

### 3.8 Motivation (Automatic, Reflective)

- **Enthusiasm for interdisciplinary collaboration**

Pharmacists consistently expressed enthusiasm for collaboration with physiotherapists, envisioning models where pharmacists are embedded in multidisciplinary teams. *– Motivation (Refl).*

*“Referral and collaboration is a huge aspect to sports medicine or sports pharmacy. I think building a close collaboration with, you know, physios or exercise phys. or whoever it may be within the local area is super important because I know where to refer if I don't know the answer.” (Pharm 1, CP, MO).*

One professional sporting context was described whereby a pharmacist was employed in a senior position within a multidisciplinary SEM team. *– Motivation (Relf).*

### 3.9 Theme.

**3: Training and Education.**

**Capability (Psychological)**

- **Gaps in undergraduate training**

A major barrier identified by many respondents was the limited emphasis on SEM in pharmacy undergraduate education. Participants consistently highlighted the insufficient training in this area. *– Capability (Psych).*

*“I think the only lecture we had or information we really had was from a one hour lecture in our for four year degree so, you know, I don't feel competent.” (Pharm 3, CP, MO).*

*“I don't often feel that pharmacists are necessarily trained in this area, or how to deal with a lot of the presentations that we do get, you know, in that kind of primary care setting that we deal with on an ongoing basis… You know, I think we probably had a matter of maybe two lectures within University.” (Pharm 1, CP, MO).*

A few respondents reported having provided education to other SEM healthcare professionals and consumers. – Capability (Psych).

*“I have been involved with actually presenting at some of the personal training sessions on medications and how they are going to influence how a person's body reacts when they get put under cardio stress.”*

*(Pharm 5, CP, MO)*

- **Preferred format of training in SEM**

A desire for hands-on training and experiential learning for practical skills like strapping and brace fitting was highlighted by some participants. *– Capability (Psych).*

*“That sort of training would have to be offered as a hands-on thing. I don't think you can really look at doing strapping or brace fitting or, you know, support fitting without being there, seeing it putting the tape on yourself knowing how it feels.” (Pharm 5, CP, MO).*

### 3.10 Opportunity (Physical)

**- Limited professional development opportunities reflecting broad scope of SEM.**

A prominent theme across the majority of interviews was that opportunities for professional development and training that reflect the broad scope of pharmacists' role in SEM are limited. *- Opportunity (Phys).*

*“Nothing has stemmed in terms of continuing professional development opportunities that, you know, I could attend.”*

*(Pharm 1, CP, MO)*

- **Opportunity for inter-professional learning**

Some pharmacists identified the opportunity for interprofessional learning in SEM, noting the potential for mutual benefits. *- Opportunity (Phys).*

*“Imagine having a pharmacist and a physiotherapist in one room learning of each other.” (Pharm 3, CP, MO).*

### 3.11 Motivation (Reflective)

- **Interest in SEM-specific professional development reflecting broad scope of SEM**

Despite the gaps in undergraduate training, all pharmacists were interested in professional development specifically tailored to SEM. *- Motivation (Refl).*

*“It's something that I would be really interested in because I certainly don't know enough about it.” (Pharm 1, CP, MO).*

One respondent employed in a professional sports setting expressed concern over the scope of a reportedly soon-to-be-released national sports pharmacy training module overlooking the broad scope of pharmacists' role in SEM, with a narrow focus on anti-doping. *– Motivation (Refl).*

*“My concern is that it's all going to be anti-doping, and I really, really hope it's not, because anti-doping is such a small amount.”*

*(Pharm 9, PS, MO)*

- **Desire for inter-professional learning**

Some pharmacists expressed a desire for collaborative learning opportunities with other health professionals, including physiotherapists. *- Motivation (Refl).*

*“I think that that's definitely something that we could be educating other health professionals about such as physios, and they perhaps could be teaching us a lot more about the practical side of things and how to deal with a sports type of injuries and how to refer appropriately.”*

*(Pharm 1, CP, MO)*

- **Preferred format of training in SEM**

Most pharmacists indicated a preference for flexible, online learning platforms tailored to SEM, including all of those practicing in a rural or remote location. *- Opportunity (Phys).*

*“Training is always better if it's online, because for rural pharmacists it's hard to get away. Training is obviously better delivered face to face because it works better, but far more likely to do training online if you're if out of any urban area.” (Pharm 8, CP, RR).*

### 3.12 Theme.

**4: Perceived barriers to Engagement in SEM.**

**Capability (Physical, Psychological)**

- **Limited skills, knowledge and resources about evidence-based non-pharmacological strategies**

Most pharmacists expressed uncertainty and lack of confidence in providing advice on non-pharmacological SEM-related queries, such as strapping techniques, joint support and equipment recommendations, due to insufficient resources and knowledge. *– Capability (Phys, Psych).*

*“I often get queries around, you know, how do I strap? What kind of strapping type do I use? I actually don't know the answer. You know, do I have to use a wrap underneath the strapping tape and those kinds of things?” (Pharm 1, CP, MO).*

*“When it comes to the more really specific sports related things, so if I was required to provide advice, like, I didn't know what to recommend to put under the tape, for example. And not only did I not know what to recommend, I also didn't know whether that was the right thing to do as to whether that would compromise the integrity of, you know, the strapping.” (Pharm 2, CM, MO).*

*“Joint support like knee guards, I'm not confident at all because really I feel like in those contexts, all I do is sort of measure the size of their joint, look at the back of the packaging, say, ‘yeah, that's the right size’. So I can help them with the product selection in a very limited way. I can talk to them about what type of support it might provide, you know, whether it's a higher support versus a lower support type fabric and style, but in terms of whether that's the right thing for them to be using in the context of their sporting needs, I'm not confident at all.” (Pharm 2, CM, MO).*

*“I'm thinking about our schedule three range of medicines where we've got really clearly defined algorithms for how we might manage that request. If we look at the flip side and we look at anything in relation to sports and recommendations around equipment, you know, or the Futuro a range of products, I mean, there's really not any sort of guidance for pharmacists, at the moment.” (Pharm 2, CM, MO).*

### 3.13 Opportunity (Physical, Social)

- **Time Constraints in Community Pharmacy**

Pharmacists frequently identified time constraints as a critical barrier to delivering SEM-related services, particularly in a community pharmacy setting. *– Opportunity (Phys).*

*“Being a home medicines review context, it was fine because I had plenty of time to look and research, etc. In a community pharmacy context, it would have been a little more difficult because I would have had to take some more time to find out what I needed.”*

*(Pharm 2, CM, MO)*

- **Lack of understanding of pharmacists' roles in SEM by other healthcare professionals**

A lack of awareness of pharmacists' expertise among other healthcare professionals was highlighted by some respondents. *- Opportunity (Soc).*

*“It's not that they were opposed, it's that they just didn't understand how a pharmacist can impact what's happening in those medical decisions.” (Pharm 9, PS, MO).*

*“I think a better understanding of what each other is capable of, and even to the point where the physio is able to refer to the pharmacy and say, ‘hey, I'd really like you to go to chat to Alison, because I think the problem we're trying to solve here is XYZ.” (Pharm 9, PS, MO).*

Regarding collaboration between pharmacists and other healthcare professionals in SEM, many participants highlighted a lack of understanding of each other's roles as a barrier to collaboration. *– Opportunity (Soc).*

*“It's not that they were opposed to pharmacist involvement, they just didn't understand how a pharmacist can impact what's happening in those medical decisions... I think there needs to be, across both professions, a better understanding of what the scope of practice is… So I think a better understanding of what each other is capable of.”*

*(Pharm 9, PS, MO)*

- **Infrequent inclusion of pharmacists in multi-disciplinary SEM teams**

Several pharmacists described limited opportunities for inclusion in multidisciplinary SEM teams as both a significant barrier and a potential untapped opportunity for enhancing patient care. *– Opportunity (Soc).*

*“I'm thinking in a more contemporary context, whether there would actually be specialised pharmacists who could work collaboratively with physios within their clinics or on sporting teams or whatever that role might be.” (Pharm 2, CM, MO).*

### 3.14 Motivation (Reflective)

- **Concerns about overstepping professional boundaries**

Some pharmacists expressed concerns about the boundaries of their role in providing advice on sports-related issues, such as mouth guards, strapping and injury recovery, and emphasised the need for clear guidelines on when to refer customers to other health professionals like physiotherapists or dentists. *- Motivation (Refl).*

*“At what point does a pharmacist, are they able to provide advice, say for example, around a mouth guard, or does that become more of like a dental type thing? Similarly around strapping and injury recovery, etc. At what point does the pharmacist kind of provide advice and at what point does the physiotherapist provide advice? So I think, you know, one of my main concerns would be around scope and pharmacists understanding, at what point are they required and should they be knowledgeable and upskilled to be able to manage this person in the pharmacy, or at what point should they be referring their customer to another health professional?”*

*(Pharm 2, CM, MO)*

- **Lack of Remuneration**

Many pharmacists practicing in community settings emphasised that adequate remuneration for their time is crucial, especially when providing detailed sports medicine advice, which often involves lengthy consultations and personalised care. *– Motivation (Refl).*

*“I guess it always comes back to the topic of remuneration, doesn't it? And, you know, to provision of advice, you know, be it self care advice or sports medicine advice or whatever that may mean, you know, adequately remunerated for pharmacists' time is a huge factor in this, particularly sports medicine which does take a lot more time when you're dealing with patient requests over the counter, for example. Sometimes it's a 20 minute or half an hour conversation.” (Pharm 1, CP, MO).*

### 3.15 Theme.

**5: Future opportunities for pharmacists in SEM.**

**Capability (Physical, Psychological)**

- **Autonomous physiotherapist prescribing**

A few pharmacists indicated their expertise in medicines use was well suited to supporting physiotherapists' prescribing, emphasising that such collaboration could enhance timely access for consumers to necessary treatments and facilitate a streamlined healthcare process. *- Capability (Psych).*

*“I do believe that professional collaboration, so that both a GP and pharmacist, kind of, you know, could assist with any problems that might arise from that prescribing activity, could be facilitated.” (Pharm 2, CM, MO).*

### 3.16 Opportunity (Social)

- **Autonomous physiotherapist prescribing**

In highlighting pharmacists' capability to support physiotherapists' prescribing, these respondents also identified this role as an opportunity for future collaboration. *- Opportunity (Soc)*

- **Improved utilisation of pharmacists' expertise in the multidisciplinary SEM landscape**

Some pharmacists identified a need for their improved integration into multidisciplinary SEM teams. *- Opportunity (Soc).*

*“Whether there would actually be specialised pharmacists who could work collaboratively with physios within their clinics or on sporting teams or whatever that role might be.” (Pharm 2, CM, MO).*

### 3.17 **Motivation (Reflective)**

- **Pharmacists are enthusiastic about playing a more meaningful role in SEM**

All pharmacists expressed enthusiasm about the growing acknowledgment of their contributions to the field of SEM. *– Motivation (Refl).*

*“I'm just excited that there's actually recognition that pharmacists belong in this this space.”*

*(Pharm 9, PS, MO)*

- **Improved utilisation of pharmacists' expertise in the multidisciplinary SEM landscape**

Some pharmacists suggested they have the knowledge and expertise to contribute meaningfully to consumer care and outcomes as part of the multidisciplinary SEM team. *- Capability (Phys, Psych).*

*“So, you know, maybe there could be something before they actually get referred to an exercise physiologist, that they could be referred to a pharmacist to provide information about the different medications.” (Pharm 5, CP, MO).*

## 4 Discussion

This study offers novel insights into Australian pharmacists' roles in contemporary SEM and their collaboration with physiotherapists. By applying the COM-B model, this research identified critical enablers and barriers influencing pharmacists' contributions to SEM, offering a foundation for future, targeted interventions to optimise their engagement within this multidisciplinary framework.

### 4.1 Key findings and contributions

#### 4.1.1 Broader scope of pharmacists in SEM

This research demonstrates that pharmacists' expertise and accessibility position them as necessary contributors to SEM. However, while much of the published literature focuses on the narrow scope of “sports pharmacy,” largely limited to doping prevention and advice on medication use in sports with minimal acknowledgment of broader applications.^10^^,^^11^^,^^20^^,^^41^, ^42^, ^43^, ^44^, ^45^ In contrast, this study highlights pharmacists' frequent engagement in SEM-related queries beyond doping control, including injury prevention and management, provision of non-pharmacological advice and products, first aid, collaboration with local sporting clubs and support for chronic disease prevention and management through physical activity. These findings suggest that pharmacists' potential in SEM extends far beyond current conceptualisations, particularly within community and multidisciplinary healthcare settings.

Some of the findings from this study are consistent with the published literature, which acknowledges pharmacists' potential role in anti-doping and willingness to counsel athletes about medicines in this context, despite concerns about knowledge and confidence in this area.^10^ However, this study also highlights the frequency and diversity of SEM-related queries encountered by pharmacists beyond these roles, ranging from holistic advice about nutrition and supplements, provision of products and information to support injury prevention and recovery to partnerships with local sporting clubs, suggestive of consumer expectation for pharmacists to provide these services. Triage and referral also emerged as key roles, consistent with other research.^46^

The findings from this study underscore the need to redefine the role of pharmacists in the context of contemporary SEM. By adopting a wider SEM perspective, pharmacists can better align their roles with the multidisciplinary approach endorsed by ACSEP and other peak bodies.^7^ Redefining this role requires targeted initiatives to enhance pharmacists' engagement in SEM, particularly in non-pharmacological interventions, injury management and interprofessional collaboration. Key steps include formalising referral pathways, integrating pharmacists into multidisciplinary SEM teams and expanding professional development opportunities beyond doping control and harnessing pharmacists' accessibility. Additionally, addressing structural barriers such as remuneration models and professional recognition are key to optimising pharmacists' contributions to SEM and improving patient care.

#### 4.1.2 Interdisciplinary collaboration in SEM

The findings of this study suggest pharmacists' collaboration with physiotherapists in SEM is as an area of untapped potential. While informal referral pathways between pharmacists and physiotherapists exist, the data suggest they lack the structure and clarity necessary for effective multidisciplinary teamwork. Strengthening these connections could improve pharmacists' integration into SEM teams, enabling more comprehensive care for individuals involved in physical activity at all levels. Enhancing referral pathways is particularly critical in SEM, where timely collaboration is essential for contexts such as managing acute sports-related injuries.

The physical environment has been shown to have a significant influence on inter-disciplinary collaboration in other disciplines, with co-location and access to resources highlighted as key factors that support clear and consistent communication between community pharmacists and general practitioners.^47^ Further, experience in collaboration encourages further collaboration, as this leads to a greater understanding of others' role and capabilities; role clarity has been identified as necessary for a multidisciplinary team to function effectively.^47^, ^48^, ^49^ Conversely, lack of understanding of each other's skills and knowledge has been shown to hinder effective interprofessional collaboration.^47^

Pharmacists' role and collaborative efforts in SEM are largely ad hoc and informal. This study found that while pharmacists may refer consumers to physiotherapists and other health professionals, these referrals are typically unstructured and lack formal pathways. Interdisciplinary collaboration in SEM remains underdeveloped, particularly in community settings, where pharmacists often make case-by-case judgments rather than working within defined referral frameworks.

In contrast, pharmacists' contributions to multidisciplinary care are well-documented in other contexts such as chronic disease, palliative care and aged care, where clear role definitions and pathways for collaboration exist.^50^, ^51^, ^52^, ^53^ However, research describing community pharmacists' interdisciplinary involvement in primary care remains limited, with much of the literature describing collaboration with physicians, particularly concerning blood pressure control.^51^^,^^54^ Similarly, published descriptions of pharmacists' collaboration in SEM are scarce and predominantly centered on competitive sports settings, overlooking their broader potential within community and multidisciplinary healthcare environments.^10^^,^^13^^,^^41^ These findings highlight the need for more structured pharmacist-physiotherapist collaboration in SEM to align with established interdisciplinary care models in other healthcare domains.

#### 4.1.3 Training and education

The findings suggest a gap in SEM-specific training for pharmacists, particularly affecting capability and opportunity. Limited undergraduate programs and professional development opportunities that reflect the full scope of pharmacists' perceived roles in SEM were key barriers, and previous research also highlights limited undergraduate training in sports pharmacy.^10^^,^^55^^,^^56^ Learning modules such as the recently launched Sports Pharmacy e-Learning course by Sports Integrity Australia are a positive step in acknowledging the role of pharmacists in SEM; however, the content of such training courses remains limited to doping prevention and medicines use and does not reflect the full scope of pharmacists' roles in SEM.^57^

Participants reported a desire for and interest in training opportunities in SEM. Pharmacists emphasised the importance of practical, hands-on training to develop skills such as strapping and brace fitting, as well as the need for flexible, online opportunities, particularly accessible to those in rural areas. Additionally, motivation for interprofessional learning was described as a key enabler for building pharmacists' engagement in SEM.

#### 4.1.4 Perceived barriers

Systemic and individual barriers were explicitly identified by participants across all three COM-B components but particularly opportunity and motivation. These included time and workload constraints in community pharmacy, limited resources to support provision of non-pharmacological advice, lack of remuneration and limited of recognition of pharmacists' roles by other healthcare professionals. These challenges limit pharmacists' ability to fully engage in SEM and highlight the need for targeted interventions to address these barriers.

#### 4.1.5 Future opportunities

Pharmacists expressed enthusiasm for expanding their roles in SEM. Interdisciplinary collaboration and supporting physiotherapists' prescribing practices were key sub-themes. Enhanced pharmacist engagement in SEM may help practitioners meet consumer expectations and enhance timely access to care, particularly when other practices may be closed.

#### 4.1.6 COM-B analysis and recommendations for future interventions

This study identified both barriers to, and enablers of pharmacists' engagement in SEM related to all three behavioural components: capability, opportunity and motivation. Addressing these challenges requires targeted interventions, and future interventions should be grounded in behaviour change theory in order to enhance the likelihood of significant and sustained behavioural change.

Future research should focus on the development of interventions that address behavioural targets such as:

- Developing SEM-specific training programs that address the full scope of pharmacists' roles in SEM may enhance *capability*. Interventions could address components of physical capability (e.g. practical skills such as strapping or first aid) or psychological capability (e.g. strategies to enhance pharmacists' knowledge and confidence in non-pharmacological SEM-related queries).
- Establishing formal referral pathways, creating opportunities for inter-professional education and advocating for pharmacists' inclusion in multidisciplinary SEM teams could enhance physical and social *opportunity* for collaboration and pharmacists' engagement in SEM.
- Promoting public awareness of pharmacists' roles in SEM while advocating for remuneration models for SEM-related consultations could enhance their reflective *motivation*, whereas automatic *motivation* could be enhanced by interventions that enhance pharmacists' personal engagement in physical activity.

### 4.2 Limitations

While this study offers valuable insights, it has several limitations that should be considered when interpreting its findings. The extended interview period (due to the researcher taking a leave of absence) may have introduced temporal bias due to changes in external factors like increased recognition of the field of sports pharmacy and publications such as the International Pharmaceutical Federation (FIP) commissioned report titled Sports pharmacy practice and education: A global overview.^58^ To mitigate this, we maintained a consistent interview guide and compared codes and themes from different periods. Retrospective analysis showed no new themes emerged after interview 8, which was undertaken earlier in the interview period.

The demographic imbalance in the interview sample, with 12 females and 2 males, may have introduced gender bias into the findings. Given that approximately 64 % of pharmacists in Australia are women,^59^ our sample overrepresents female perspectives. Additionally, many participants were from metropolitan areas, predominantly New South Wales, which may limit the transferability of our results. These factors should be considered when interpreting the findings, and future research should aim for a more balanced and diverse participant pool.

Participants predominantly practiced in community settings. While community settings are where many SEM-related presentations occur, this may restrict the transferability of the results to other practice environments such as hospitals, and may underrepresent male perspectives. Additionally, the study captures only the perceptions of pharmacists, excluding valuable insights from consumers and other stakeholders such as sports coaches and other health professionals, including physiotherapists, that could provide a more comprehensive understanding of interprofessional collaboration as well as consumer and organisation needs. While the study effectively applies the COM-B framework to explore pharmacists' roles and behaviours, it does not address broader systemic barriers, such as healthcare policies and funding limitations. The focus on the Australian healthcare context further limits applicability to international settings, as evidenced by differences highlighted in the systematic review, which included data from other countries including Qatar, the USA and Canada. Lastly, the reliance on self-reported data introduces the potential for bias, as participants' perceptions may not fully reflect their actual practices or the complete range of barriers and enablers affecting their engagement in SEM.

### 4.3 Context

This study's findings align with the broader vision of SEM, which aims to provide for safe and effective physical activity for all members of the community. Expanding the role of pharmacists in SEM could have significant public health benefits, including reducing the burden of chronic diseases and improving access to care. This study provides a foundation for developing evidence-based interventions to optimise pharmacists' engagement in SEM. This study constituted the first phase of a broader research initiative to inform the development of an intervention toolkit to optimise pharmacists' engagement in SEM, including pharmacist-physiotherapist collaboration as part of the multidisciplinary SEM team.

## 5 Conclusion

This research uniquely contributes to the understanding of pharmacists' roles in SEM and their collaboration with physiotherapists. By addressing identified barriers and leveraging enablers, we can enhance pharmacists' engagement in SEM, improve multidisciplinary care and ultimately promote better health outcomes. Future research should focus on developing and evaluating interventions, grounded in behaviour change theory, that build pharmacists' capability, opportunity, and motivation to fulfill their potential in this emerging area of healthcare.

## CRediT authorship contribution statement

**Alison D. Hooper:** Writing – review & editing, Writing – original draft, Visualization, Validation, Project administration, Methodology, Investigation, Formal analysis, Data curation, Conceptualization. **Jodie Marquez:** Writing – review & editing, Visualization, Supervision, Methodology, Conceptualization. **Beata Bajorek:** Writing – review & editing, Visualization, Supervision, Methodology, Conceptualization. **Joyce Cooper:** Writing – review & editing, Visualization, Supervision, Methodology, Conceptualization. **David Newby:** Writing – review & editing, Visualization, Supervision, Methodology, Conceptualization.

## Funding

This research is supported by an Australian Government Research Training Program (RTP) Scholarship.

## Declaration of competing interest

**Alison Hooper.**

Alison is an employee of the University of Newcastle. The research is funded under the Australian Government's Research Training Program (RTP).

**Jodie Marquez.**

I have nothing to declare.

**Beata Bajorek.**

I have nothing to declare.

School of Biomedical Sciences and Pharmacy, University of Newcastle, Australia.

Hunter Medical Research Institute, Australia.

Hunter New England Local Health District, Australia.

**Joyce Cooper.**

I have nothing to declare.

**David Newby.**

I have nothing to declare.

## Figures and Tables

**Table 1:** Semi-Structured Interview Guide.

Category	Questions (approximate time allocated)	Prompts
**Introduction**	I appreciate your participation in this interview and value your perspective on sports pharmacy and pharmacist-physiotherapist collaboration in sport and exercise medicine. It's important to emphasise that there are no right or wrong answers; we are solely interested in hearing your opinion. Thank you for sharing your insights. The interview will be recorded as previously advised. Do you have any questions about the research before we begin? (1–2 min)	**–**
**Demographic questions**	In which Suburb/State do you practice? How many years have you spent as a registered pharmacist? In what setting/s do you practice? (1–2 min)	**–**
**Interview guide**	What comes to mind when you hear the term ‘Sports Pharmacy’? (8–10 min) In your role as a pharmacist, what health queries have you received that you feel would fall into the category of ‘sports pharmacy’? (8–10 min) *(If the participant has not received any, ask them to discuss why they feel this is the case)* What aspects of sports-related health care do you feel pharmacists could play a greater or more meaningful role in the future? (+/− 5 min) In your experience or opinion, how might collaboration between pharmacists and physiotherapists contribute to enhancing the practice of sport and exercise medicine? (+/− 5 min)	**Capability (Physical, Psychological)** Does responding to sports-related health queries require any particular knowledge or skills? (PsycC, PhysC) - Tell me more about how this influences your practice of sports pharmacy. **Opportunity (Physical, Social)** How does your practice environment influence your practice of, or ability to practice, sports pharmacy? (PO) • Time, resources? • To what extent do you feel you have the necessary resources to practice sports pharmacy? How does your social environment influence your practice of, or ability to practice, sports pharmacy? (SO) • How does inter-professional collaboration influence your practice of, or ability to practice, sports pharmacy? • How do you collaborate with physiotherapists in practicing sports pharmacy? How do you feel this would support the broader practice of sport and exercise medicine? What impact do you think improved opportunities for pharmacist-physiotherapist collaboration would have on patient care in sport and exercise medicine? (SO) • Referral, sharing of knowledge and resources. • Appropriate non-pharmacological advice, safe and effective use of medicines such as analgesics or anti-inflammatories. • How do you envisage this occurring? **Motivation (Reflexive, Automatic)** How important do you feel it is to provide sports pharmacy services? (RM) How do you feel about providing sports pharmacy services? (AM) In your experience, can you share how your personal engagement in or enthusiasm for physical activity may impact your approach to sports pharmacy? (AM)

PhysC: Physical Capability; PsycC: Psychological Capability; PO: Physical Opportunity; SO: Social Opportunity; RM: Reflective Motivation; AM: Automatic Motivation.

**Table 3:** Themes and sub-themes identified as influencers of pharmacists' engagement in SEM and pharmacist-physiotherapist inter-professional collaboration, mapped to COM-B components.

Theme	Sub-Theme	COM-B Component⁎	Enabler/Barrier/Both
Broad scope of pharmacy practice in SEM incorporating both pharmacological and non-pharmacological advice	Pharmacists as medicines experts	Capability (Phys, Psych)	Enabler
	Frequent SEM-related queries	Opportunity (Phys, Soc)	Enabler
	Broad scope of queries in SEM	Capability (Phys, Psych); Opportunity (Phys, Soc)	Both
	Partnerships with local sporting clubs	Opportunity (Soc)	Enabler
	Diverse demographic of consumers across the lifespan	Opportunity (Phys)	Enabler
	Pharmacists' accessibility supports their role in SEM	Opportunity (Phys); Motivation (Refl)	Enabler
	Consumer expectations	Opportunity (Soc); Motivation (Refl)	Both
	Pharmacists more likely to engage in SEM if they have a personal interest in sports	Motivation (Auto)	Both
	Acting as a triage point	Capability (Psych); Opportunity (Phys)	Enabler
Opportunities and challenges in interprofessional collaboration in SEM	Pharmacists often suggest consumers see a physiotherapist	Opportunity (Soc)	Enabler
	Informal referral pathways	Opportunity (Phys, Soc)	Both
	Enthusiasm for interdisciplinary collaboration	Motivation (Refl); Opportunity (Phys)	Enabler
	Medicine requests post-physiotherapist consultation	Opportunity (Soc); Capability (Psych)	Both
Training and education	Gaps in undergraduate training	Capability (Psych)	Barrier
	Limited professional development opportunities reflecting broad scope of SEM	Opportunity (Phys)	Barrier
	Interest in SEM-specific professional development reflecting broad scope of SEM	Motivation (Refl)	Enabler
	Opportunity for inter-professional learning	Opportunity (Phys)	Enabler
	Desire for inter-professional learning	Capability (Psych); Motivation (Refl)	Enabler
	Preferred format of training in SEM	Capability (Psuch); Opportunity (Phys)	Both
Perceived barriers to engagement in SEM	Time constraints in community pharmacy	Opportunity (Phys)	Barrier
	Limited skills, knowledge and resources about evidence-based non-pharmacological strategies	Capability (Phys,Psych)	Barrier
	Lack of understanding of pharmacists' roles in SEM by other healthcare professionals	Opportunity (Soc)	Barrier
	Concerns about overstepping professional boundaries	Motivation (Refl)	Barrier
	Infrequent inclusion of pharmacists in multi-disciplinary SEM teams	Opportunity (Soc)	Barrier
	Lack of Remuneration	Motivation (Refl)	Barrier
Future opportunities for pharmacists in SEM	Autonomous physiotherapist prescribing	Capability (Psych); Opportunity (Soc)	Enabler
	Pharmacists enthusiastic about playing a more meaningful role in SEM	Motivation (Refl)	Enabler
	Improved utilisation of pharmacists' expertise in the multidisciplinary SEM landscape	Capability (Psych); Opportunity (Soc)	Enabler

⁎ Capability: Phys = Physical, Psych = Psychological. Opportunity: Phys = Physical, Soc = Social. Motivation: Auto = Automatic, Refl = Reflective.

## Data Availability

Due to the sensitive nature of the questions in the interviews, respondents were assured the raw data would remain confidential and respondents would not be identifiable.

## Glossary

**Sport and Exercise Medicine (SEM)**: A multidisciplinary healthcare discipline that focuses on optimising sporting performance at all levels while promoting physical activity for the prevention and management of chronic diseases such as arthritis, diabetes and cardiovascular disease. SEM extends beyond competitive athletes to include recreationally active individuals and those who can benefit from increased physical activity for health and disease prevention.
**Sports Pharmacy**: A sub-discipline of pharmacy whose description to date largely involves the provision of medicines-related advice and support to athletes and physically active individuals, including anti-doping regulations and supplement use.
**COM-B Model**: A theoretical framework used to understand behaviour, identifying three key components: Capability (physical and psychological), Opportunity (social and physical), and Motivation (reflective and automatic), which interact to influence behaviour.
**Behaviour Change Wheel (BCW)**: A theoretical framework that builds on the COM-B model, used to design interventions aimed at influencing health-related behaviours.
**Anti-Doping**: The set of regulations, policies and practices designed to prevent the use of performance-enhancing drugs and other prohibited substances in competitive sports.
**Triage**: The process of assessing and prioritising patient care based on urgency, which may incorporate referral, particularly in primary healthcare settings where pharmacists may refer individuals to other health professionals.
**Referral Pathways**: Formalised processes that guide healthcare providers in directing patients to appropriate healthcare providers or services for further assessment or treatment.
**Non-Pharmacological Interventions**: Treatments that do not involve medicines, such as exercise, dietary advice, mobility aids and the provision of straps or supports, which pharmacists may provide in the course of their practice.
